# A network-centric approach reveals novel pathways impacted by Prader-Willi Syndrome

**DOI:** 10.1371/journal.pone.0347773

**Published:** 2026-04-28

**Authors:** Kunal Bham, Manju Anandakrishnan, Cathy H. Wu, Karen E. Ross

**Affiliations:** 1 Department of Biochemistry and Molecular and Cellular Biology, Georgetown University Medical Center, Washington, District of Columbia, United States of America; 2 Center for Bioinformatics and Computational Biology, University of Delaware, Newark, Delaware, United States of America; Nathan S Kline Institute, UNITED STATES OF AMERICA

## Abstract

Prader-Willi Syndrome (PWS), a rare multi-system disorder characterized by insatiable appetite, growth abnormalities, and cognitive delay, results from genetic defects in a paternally expressed region of chromosome 15, q11.2-q13. This region contains several protein-coding genes and several genes encoding small nucleolar RNA (snoRNAs), including the *SNORD116* gene cluster, but their exact role in PWS remains unclear. Since snoRNAs have wide-ranging effects on protein expression and proteins interact in a complex network, the genetic aberrations causing PWS are likely to cause far-reaching indirect effects on protein expression and activity. Here, we mapped PWS gene expression data onto a human protein-protein interaction (PPI) network and used graph learning techniques to 1) identify the most impacted proteins and 2) suggest novel disease mechanisms. We adapted GeneEMBED, a network-based method originally developed to model genetic variants associated with Alzheimer’s Disease. Specifically, we integrated PWS or control expression data with the PPI network, calculated node embeddings, and identified proteins with large differences between PWS and control embeddings. These candidate proteins were subjected to functional enrichment analysis to discover altered biological processes in PWS. Candidate proteins were highly enriched for glycosylated proteins. Analysis of candidate glycosylation enzymes suggested abnormalities in mucin-type O-glycosylation, fucosylation, and glycosaminoglycan synthesis. Defects in these glycosylation pathways have been linked to several PWS phenotypes, including obesity, cognitive delay, and production of secondary sex hormones. Homeobox proteins, master regulators of transcription during development, were also overrepresented among the candidate proteins. In particular, we identified homeobox proteins that drive development of GABAergic and dopaminergic neurons. These neuronal pathways regulate appetite and other behaviors that are abnormal in individuals with PWS. Our results were highly reproducible across PWS model systems. This work offers new avenues for further research in PWS and provides a promising approach that can be applied to other complex diseases.

## Introduction

Prader-Willi Syndrome (PWS) is a rare genetic disorder caused by the loss of function of specific genes on chromosome 15 that affects endocrine, metabolic, and neurologic systems [1]. Key symptoms of PWS include hyperphagia, obesity, cognitive delay, growth hormone deficiency, hypogonadism, and skeletal abnormalities [2]. PWS is the most common genetic cause of morbid obesity in children [3], occurring in approximately 1 in 10000–1 in 25000 births [4]. Currently, there is no cure for PWS, in part because of the limited understanding of the specific contributions of individual genes to the overall PWS phenotype.

PWS is characterized by a genetic abnormality in an imprinted, paternally expressed region of chromosome 15, q11-q13. In most PWS cases, this region of the paternally inherited allele carries a deletion of several megabases [5]. The deleted region includes protein-coding genes (including MKRN3, MAGEL2, and NDN), several species of small nucleolar RNAs (SNORD65, SNORD108, two copies of SNORD109, 30 copies of SNORD116, and 48 copies of SNORD115), antisense RNA that can silence UBE3A (UBE3A-ATS), and other less well-understood RNA species [6–10].

Studying the association between these genes and the PWS phenotype is crucial to understanding the disease’s molecular mechanism. Many prior studies have performed differential gene expression analysis comparing cell lines from individuals with PWS to control individuals [11–14]. However, protein-level studies are far less common, leaving critical gaps in our understanding of protein interaction networks. Since proteins interact in a complex network, changes in the expression or activity of one protein can have far-reaching and unexpected effects even on indirectly connected proteins. Therefore, considering protein interaction networks along with differential gene expression data may offer valuable insights into the molecular mechanistic links and the disease phenotype generally not addressed by most disease-gene association methods.

One way to analyze protein interaction networks is through knowledge graph representation. Knowledge graph representation offers an opportunity for disease analysis in the context of biological networks [15,16]. Embedding, a technique to represent complex data such as text, images, or networks, as low-dimensional vectors, has gained popularity and is now used widely in biomedical research [17,18]. Specifically, node embedding compresses the qualitative and quantitative characteristics of each node in a network into computationally tractable vectors [19]. The GraphWave node embedding algorithm produces similar embeddings for nodes in similar local network environments [20]; therefore, if GraphWave embeddings are calculated for two networks with the same structure (i.e., nodes and edges) but different edge weights, differences in a node’s embeddings between the two networks will reflect the contribution of the edge weights only. Furthermore, if the edge weights for two networks are calculated using data from disease vs. healthy samples, differences in node embeddings may provide insight into the disease pathology. The GeneEMBED method exploits this approach by integrating gene variant data from Alzheimer’s patients into protein-protein interaction (PPI) networks to identify disease mechanisms [21]. Here, we adopt a similar framework by mapping PWS gene expression data onto a human PPI network from the STRING database [22] to identify the proteins most impacted, directly or indirectly, by the PWS genetic defect. These differences would not necessarily be visible through typical differential expression analysis, which considers only the expression value of individual genes in isolation.

## Results

### Identification of genes affected by a large deletion in the PWS locus

To identify proteins affected, directly or indirectly, by gene expression changes in PWS cells, we first used gene expression data from neuronal cells derived from the human embryonic stem cell line, CT2, with and without a large deletion in the PWS region on chromosome 15 [23]. The expression values were overlaid as edge weights on PPI networks from STRING to create two weighted PPI networks: CT2-lgDel and CT2-Control. To interpret these data in a network context we adapted the GraphWave-based embedding framework. Since the network structure is held constant, differences in node embeddings between conditions reflect changes driven by edge weights. These embedding differences represent how each protein’s network context is altered between PWS and control, thus capturing proteins affected by both direct and indirect effects of the PWS-associated gene expression changes.

**Fig 1:**
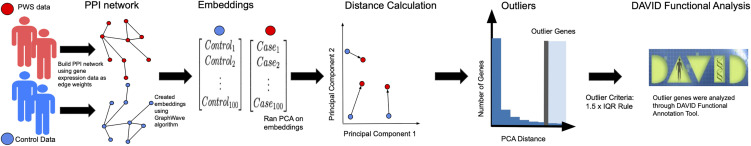
Analysis workflow diagram with steps for (1) Data Preprocessing, (2) Building PPI Networks, (3) Generating Node Embeddings, (4) Distance Calculation, (5) Outlier Detection, (6) Enrichment Analysis.

Applying the network embedding-based workflow outlined in Fig 1 (see Methods), we identified 2002 outlier proteins out of 15980 total proteins. Next, to identify potential false positives, we compared two CT2-Control networks (CT2-Control-A and CT2-Control-B; see Methods), and found 102 outlier proteins. There were six outlier proteins in common between the two analyses. These were removed from the CT2-lgDel vs. CT2-Control outlier set, yielding a final set of 1996 proteins, which we refer to as CT2-lgDel candidate proteins (Tables S1 and S2 in S1 File).

**Fig 2:**
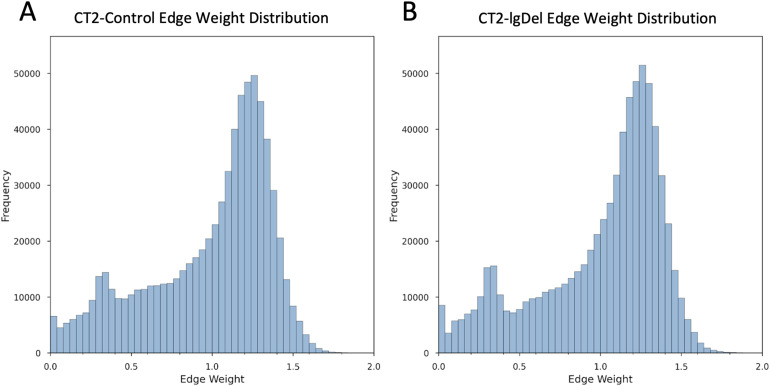
Frequency histogram showing the distribution of network edge weights after rescaling to a range of 0-2 for (A) CT2-Control, (B) CT2-lgDel.

Before proceeding with the biological interpretation of the results, we performed several control analyses to validate our approach. As shown in Fig 2, the distributions of input edge weights in the CT2-Control and CT2-lgDel networks exhibit a similar pattern, indicating that overall gene expression patterns were similar in the Control and the lgDel subgroups.

**Fig 3:**
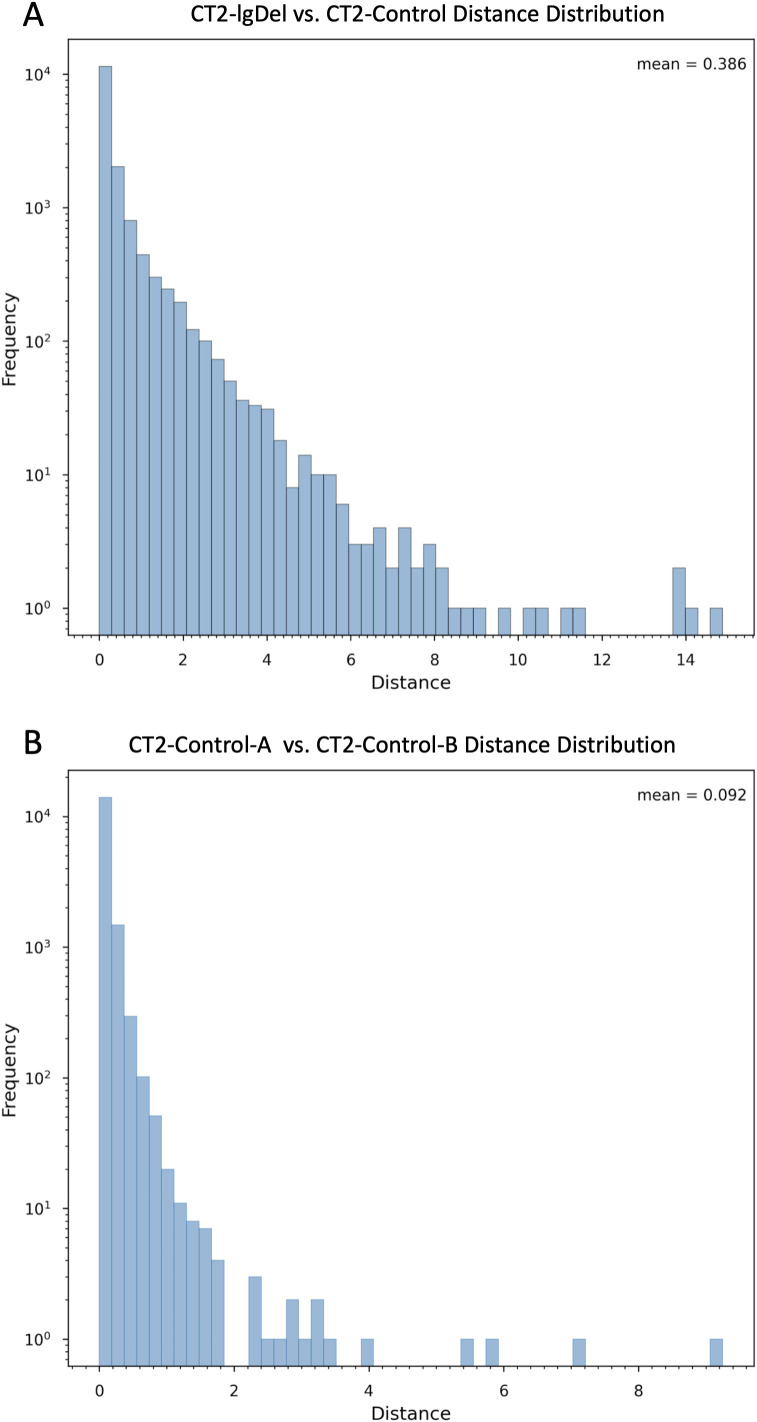
Log-scaled frequency histogram showing the distribution of Euclidean distances between protein nodes in (A) CT2-lgDel vs. CT2-Control networks and (B) CT2-Control-A vs. CT2-Control-B networks.

Next, we examined the distribution of distance values obtained in the CT2-lgDel vs. CT2-Control (Fig 3A) and CT2-Control-A vs. CT2-Control-B (Fig 3B) analyses. In the CT2-lgDel vs. CT2-Control analysis, most proteins exhibit distances near zero, indicating minimal changes for the majority of proteins, as expected. However, we observed a substantial number of proteins with distances between two and eight and a significant right tail, with distances for a few proteins greater than 14 (Fig 3A). Overall, distance values in the CT2-Control-A vs. CT2-Control-B analysis are much lower (mean = 0.092 vs. mean = 0.396 for the lgDel-Control analysis; Fig 3B). Most proteins have distances near zero and very few have distances greater than two. This is consistent with our expectation that proteins would not exhibit large distances in a comparison of genetically identical replicates.

To ensure that gene expression levels did not introduce bias into our distance measurements, Pearson correlation coefficients were calculated between the average gene expression levels of proteins and their respective distance values. In the CT2-lgDel vs. CT2-Control analysis, the correlation coefficient was near-zero (r = −0.07969); however, in the CT2-Control-A vs. CT2-Control-B analysis, the correlation was moderately positive (r = 0.63065) (Table S1 in S1 File). The lack of a strong correlation in the lgDel vs. Control comparison suggests that the observed distances are not merely driven by baseline gene expression but instead likely reflect functional changes in the network caused by the deletion within the PWS locus. The positive correlation observed in the Control-A vs. Control-B comparison indicates that proteins with higher gene expression levels tend to have higher distances. This correlation suggests that our method for identifying false positives is effectively capturing proteins whose distances are primarily influenced by gene expression levels rather than true disease-driven network disruptions.

Finally, we observed a near-zero Pearson correlation (r=−0.082) between the protein-wise distances in the lgDel/Control vs. the Control-A/Control-B analyses, highlighting that lgDel/Control distances are primarily driven by PWS-specific network effects rather than underlying structural properties of genes in the network.

### Links between candidate genes and known PWS-associated genes

To further validate our results, we looked for overlap between the CT2-lgDel candidate proteins and 17 protein-coding genes located within the PWS locus on chromosome 15 (Table S3 in S1 File; [24]). Eight genes/proteins—SNRPN, NDN, MAGEL2, MKRN3, SNURF, NPAP1, OCA2, and ATP10A—were found in both sets. A Fisher’s exact test revealed that the observed overlap of eight genes from the PWS region is significantly higher than expected by chance (Odds Ratio: 6.22; p-value = : 5.15e-04). This indicates a strong enrichment of genes from the PWS region in our candidate protein set, supporting the validity of the method.

In the original analysis of this dataset, the authors identified 40 protein-coding genes that were consistently transcriptionally dysregulated in all PWS genetic models and cell lines examined (Table S4 in S1 File; [23]). Upon comparison, we found our CT2-lgDel candidate list included eight of these 40 proteins: MAGEL2, SYK, HSPA1L, PAX6, IL1RAPL1, COL18A1, MYBL2, CDH20. While the degree of overlap was not significant (Fisher’s Exact Test: Odds Ratio: 1.75; p-value = 0.15), this overlap may further support the biological significance of these genes to PWS. Only one of the overlapping proteins, MAGEL2, is encoded in the PWS region; the others are affected indirectly by the PWS deletion.

Thus, while our CT2-lgDel candidate list is enriched for proteins with a known association with PWS, it also contains many novel genes that are neither located in the PWS genetic region nor were identified through traditional differential gene expression analysis.

### Reproducibility of candidate genes across cell lines

**Fig 4:**
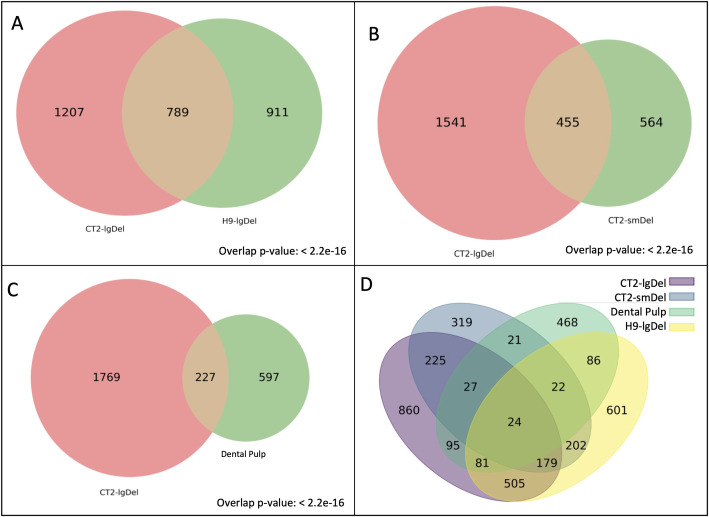
Venn Diagrams showing overlap in candidates genes identified in (A) CT2-lgDel and H9-lgDel, (B) CT2-lgDel and CT2-smDel, (C) CT2-lgDel and Dental Pulp, (D) all four analyses.

We repeated our network embedding analysis using gene expression data from neurons derived from another human embryonic stem cell line, H9, with an identical large deletion of the PWS region [23]. After removal of potential false positives, we identified 1,700 candidate proteins (H9-lgDel candidates), with 789 overlapping with the CT2-lgDel candidates (Tables S1 and S5 in S1 File; Fig 4A). The overlap is very highly significant (Fisher’s Exact Test: Odds Ratio: 9.38; p-value < 2.2e-16). Moreover, the H9-lgDel candidate list included six proteins encoded in the PWS locus (SNRPN, NDN, MAGEL2, MKRN3, SNURF, and NPAP1), all of which were also CT2-lgDel candidate genes, and ten proteins encoded by genes that are transcriptionally dysregulated in PWS according to Gilmore et al. [23] (IL1RAPL1, CDH20, PAX6, HSPA1L, MAGEL2, SYK, MYBL2, PAX5, KIF24, RECQL4), seven of which are also CT2-lgDel candidate proteins (Tables S3 and S4 in S1 File). These results demonstrate that our method is robust and highly reproducible across cell lines with distinct genetic backgrounds harboring the same engineered deletion in the PWS locus.

### Candidate genes are highly enriched for glycosylated proteins

The CT2-lgDel candidate proteins were analyzed using DAVID to identify significantly enriched functional terms. These terms were examined individually and grouped into clusters based on gene co-occurrence. Fifty-three clusters were identified as significant (enrichment score ≥ 2). Table 1 highlights the most significant clusters (for complete results see Table S6 in S1 File). The top enriched term was glycoprotein (Benjamini-Hochberg corrected p-value = 2.15e- 56); 747 out of the 1996 candidate genes were annotated with this term. The most enriched cluster contains the term glycoprotein along with the related UniProt Sequence Feature CARBOHYD:N-linked (GlcNAc…) Asparagine [25]. Moreover, several other clusters in the top five include a high proportion of glycosylated proteins: Secreted Components (73%), Extracellular Matrix (87%), Membrane Components (63%), and Ion Transport (52%) (Table 1).

**Table 1:** CT2-lgDel functional enrichment clusters.

Cluster Name	Top Biological Functions	Enrichment Score	Total Proteins*	Glycosylated Proteins**	% Glycosylated
Glycoprotein	Glycoprotein, CARBOHYD:N-linked (GlcNAc..) Asparagine, Disulfide Bond, Signal	40.43	1006	747	74
Secreted Proteins	Secreted Proteins, Extracellular Space and Region	31.68	711	518	73
Extracellular Matrix	ECM Organization, Matrix Components	18.16	115	100	87
Membrane Components	Plasma Membrane, Transmembrane Helices, Cell Membrane	16.04	972	505	52
Ion Transport	Ion Transport, Ion Channel Activity	9.84	129	81	63
Homeobox	Homeobox, HOX	8.67	62	0	0

* Number of unique proteins associated with at least one term in the cluster.

** Number of unique proteins associated with at least one term in the cluster that are annotated with the UniProt keyword Glycoprotein (KW-0325).

A similar DAVID analysis of the H9-lgDel candidate proteins revealed 43 significant clusters (enrichment score ≥ 2), of which 28 clusters overlapped with the CT2-lgDel clusters (Table 2, Table S7 in S1 File). The top two H9-lgDel clusters consisted of terms related to glycosylation, membrane proteins, and extracellular proteins, again pointing to the strong enrichment of glycosylated proteins among the candidate proteins.

**Table 2:** H9-lgDel functional enrichment clusters.

Cluster Name	Top Biological Functions	Enrichment Score	Total Proteins*	Glycosylated Proteins**	% Glycosylated
Extracellular	Secreted Proteins, Extracellular Space and Region, Signal	23.41	610	455	75
Glycoprotein and Membrane	Glycoprotein, N-linked Asparagine, Disulfide Bond, Signal, Plasma Membrane, Transmembrane Helices, Cell Membrane	16.99	1018	632	62
Homeobox	Homeobox, HOX	11.85	67	1	1
Transcription	Sequence-specific Double Stranded DNA Binding, Positive Regulation of Transcription by RNA Polymerase II, Chromatin, DNA Binding Transcription Factor Activity	8.29	323	40	12
Development	Developmental Protein, Differentiation	7.27	180	46	26
Vision	Vision, Visual Perception, Sensory Transduction	6.29	51	13	25

* Number of unique proteins associated with at least one term in the cluster.

** Number of unique proteins associated with at least one term in the cluster that are annotated with the UniProt keyword Glycoprotein (KW-0325).

To further explore potential changes in glycosylation in our PWS models, we compared the lists of CT2-lgDel and H9-lgDel candidate proteins to a list of ~350 glycosylation enzymes [26]. Twenty-eight of the CT2-lgDel candidate genes and 23 of the H9-lgDel candidate genes were glycosylation enzymes (Table S8 in S1 File). Twelve of these enzymes (B3GNT6, B4GALNT2, CHST13, CHST4, FUT2, FUT3, FUT5, GALNT5, GCNT3, HAS2, UGT2B15, UGT2B7) were candidates in both cell lines (Fisher’s Exact Test for overlap: Odds Ratio: 24.85; p-value = 1.3e-09). Heatmaps of the expression levels of the candidate glycosylation enzymes in each cell line are shown in S1 Fig. While there are differences in the expression pattern between control and PWS cells for many of the enzymes, only nine (out of 28) of the CT2 candidate enzymes and three (out of 23) of the H9 candidate enzymes are significantly differentially expressed (p adj. < 0.05). This illustrates the point that candidate genes may not necessarily be highly differentially expressed in PWS cells, but may still play an important role in the disease state due to indirect effects caused by changes in expression of neighboring genes in the network.

**Fig 5:**
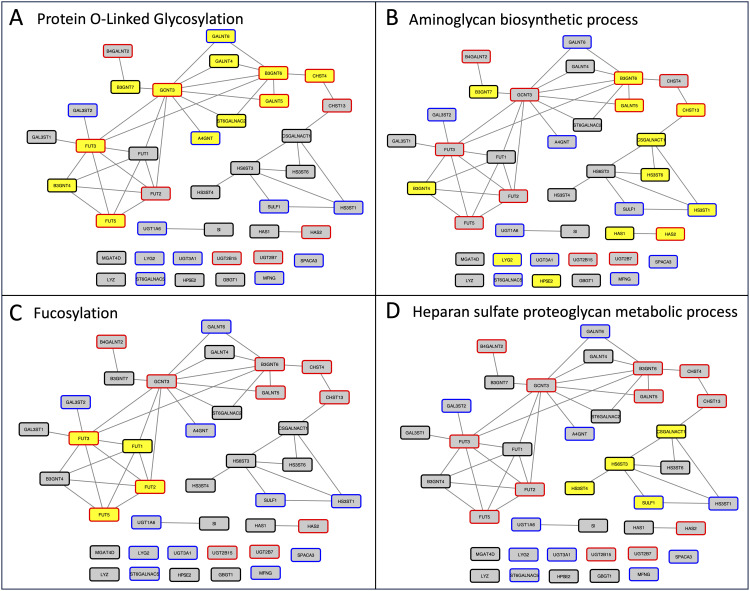
Network showing the protein-protein interactions among the glycosylation enzymes that are CT2-lgDel and/or H9-lgDel candidates. Nodes with black borders are CT-lgDel candidates; nodes with blue borders are H9-lgDel candidates; nodes with red borders are both CT2-lgDel and H9-lgDel candidates. Yellow node fill color indicates the protein is annotated with the following enriched GO Biological Process terms: **(A)** Protein O-linked glycosylation (GO:0006493; FDR for enrichment in the network = 9.57e-16); **(B)** Aminoglycan biosynthetic process (GO:0006023; FDR = 5.24e-13); **(C)** Fucosylation (GO:0036065; FDR = 3.06E-05); **(D)** Heparan sulfate proteoglycan metabolic process (GO:0030201; FDR = 1.6e-04).

STRING protein-protein interactions among the candidate glycosylation enzymes are shown in Fig 5. A connected subgroup of twelve enzymes, including six of the twelve enzymes in common between the CT2-lgDel and H9-lgDel candidate lists, participates in O-glycosylation (Fig 5A). Other subgroups of enzymes participate in fucosylation, aminoglycan biosynthesis, and heparan sulfate proteoglycan metabolism (Fig 5B-5D).

**Fig 6:**
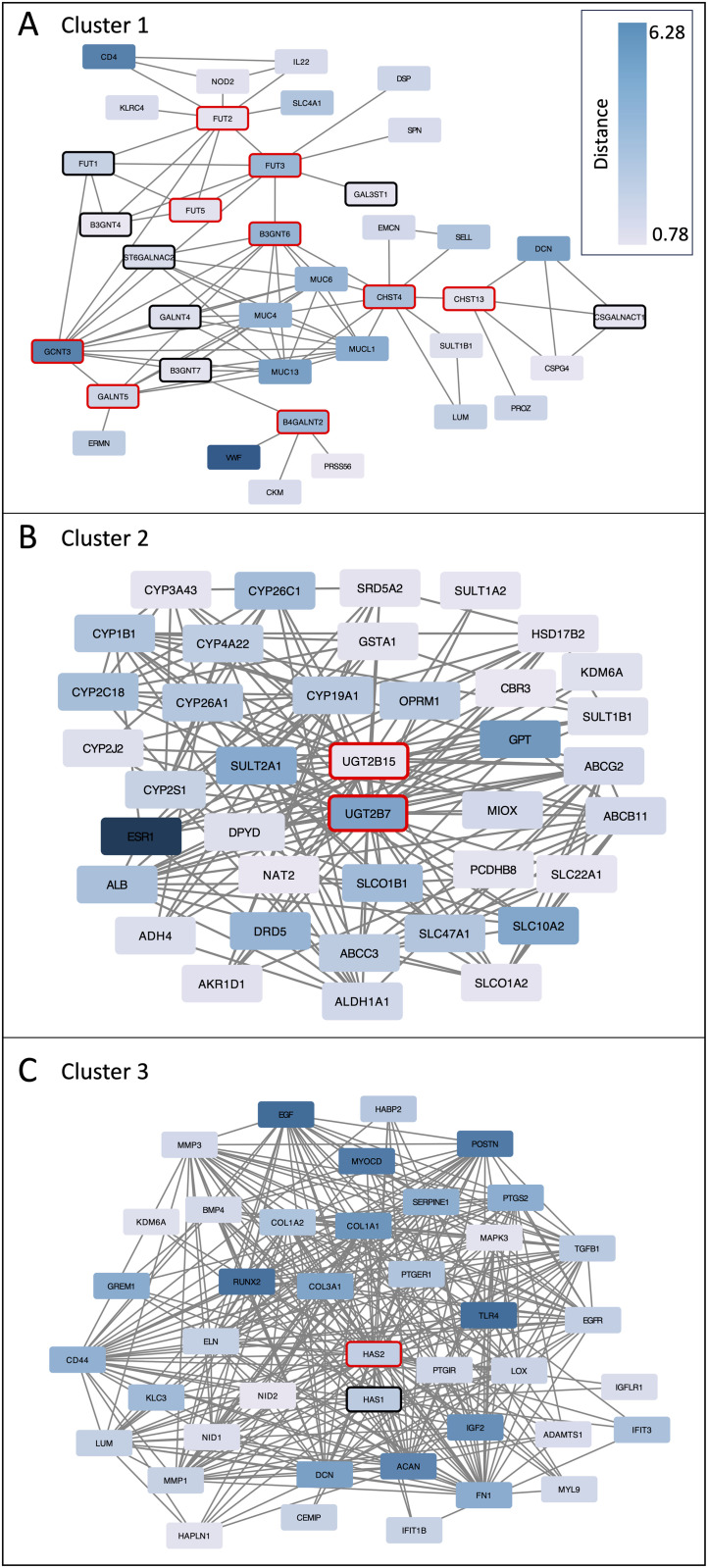
Networks showing protein-protein interactions among CT2-lgDel candidate glycosylation enzymes and their CT2-lgDel candidate first neighbors. Nodes with black borders are glycosylation enzymes that are CT2-lgDel candidates; nodes with red borders are glycosylation enzymes that are both CT2-lgDel and H9-lgDel candidates. Node fill color indicates Euclidean distance between lgDel and control networks. The network clustered into three sub-networks centered around different subsets of glycosylation enzymes. Clusters 1-3 shown in **(A)-(C)**, respectively.

Next, we examined the STRING protein-protein interactions among the CT2-lgDel candidates, focusing on the twelve glycosylation enzymes that were candidates in both cell lines and their first neighbors (Fig 6; S2 Fig). The network consists of three distinct clusters. Cluster 1 (Fig 6A) includes nine of the twelve enzymes (B3GNT6, B4GALNT2, CHST13, CHST4, FUT2, FUT3, FUT5, GALNT5, GCNT3). The first neighbors include several mucin (MUC4, MUC6, MUC13) and mucin-like (MUCL1) proteins, which are heavily O-glycosylated, and the enriched functional terms for the proteins in this cluster are mainly related to O-glycosylation. Cluster 2 (Fig 6B) contains two glycosylation enzymes–UGT2B7 and UGT2B15–which are UDP-glucuronosyltransferases required for attachment of glucuronic acid to lipophilic substrates including steroid hormones and some drugs [27]. Several of the first neighbors are cytochrome p450 enzymes, which are also essential for drug and steroid hormone metabolism. Consistent with these results, the top enriched function terms for this cluster include steroid hormone biosynthesis, xenobiotic metabolism, and retinol metabolism. Finally, Cluster 3 (Fig 6C) is centered on HAS2, which participates in the synthesis of hyaluronan, a glycosaminoglycan (GAG) that is an essential component of the extracellular matrix especially in connective tissue. Taken together, our results indicate that PWS cells may have abnormalities in specific aspects of glycosylation, namely O-glycosylation, fucosylation, and production of GAGs (heparan sulfate and hyaluronan).

### Candidate genes are highly enriched for homeobox proteins

**Fig 7:**
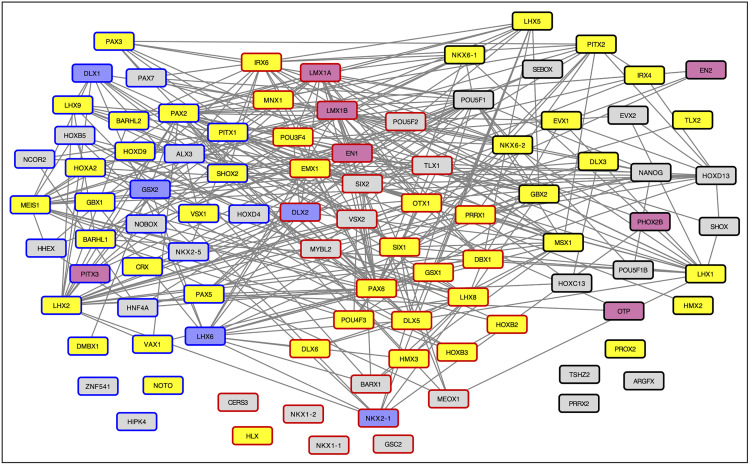
Network showing the protein-protein interactions among the proteins from the DAVID Homeobox cluster that are CT2-lgDel and/or H9-lgDel candidates. Nodes with black borders are CT-lgDel candidates; nodes with blue borders are H9-lgDel candidates; nodes with red borders are both CT2-lgDel and H9-lgDel candidates. Node fill color indicates GO Biological Process annotations: Yellow: Nervous system development (GO:0007399; FDR for enrichment in the network = 4.34E-32; Pink: Dopaminergic neuron differentiation (GO:0071542; FDR = 1.22E-07; Purple: GABAergic neuron differentiation (GO:0097154; FDR = 4.83E-06).

Another highly enriched cluster in the functional enrichment analysis of both the CT2-lgDel and H9-lgDel candidates consisted of homeobox proteins, a class of transcription factors that are involved in differentiation and development (Tables 1 and 2). Ninety-five proteins from the CT2-lgDel and/or H9-lgDel candidate lists were associated with at least one term in the homeobox cluster, and 34 of the homeobox proteins were candidates in both CT2-lgDel and H9-lgDel (Fig 7). The STRING protein-protein interactions among the homeobox candidates are shown in Fig 7. Most of the proteins (82/95) form a single highly connected cluster. About two-thirds of the proteins (61/95) are associated with nervous system development (Fig 7 yellow, purple, and pink-filled nodes). Moreover, the homeobox proteins are enriched for factors involved in the differentiation of GABAnergic (Fig 7, purple nodes) and dopaminergic neurons (Fig 7, pink nodes), two classes of neurons that have been implicated in PWS symptoms (see Discussion).

### Generalizability of results to other PWS expression datasets

Although many people with PWS have large chromosome 15 deletions similar to the CT2-lgDel and H9-lgDel models, some have smaller atypical deletions centered on the SNORD116 locus. We were interested to see whether similar proteins and processes would be affected in a cell line that models this smaller deletion. Therefore, we repeated our analysis using public RNAseq data from CT2 cells containing a deletion that targets the SNORD116 locus only [23]. This dataset (CT2-smDel) revealed 1,019 candidate proteins (Table S9 in S1 File), 453 of which overlapped with those identified in the CT2-lgDel analysis (Fig 4B; Fisher’s Exact Test on overlap: Odds Ratio: 6.59; p-value < 2.2e-16). Using DAVID functional enrichment analysis, we found 23 clusters with enrichment scores > 2 (Table S10 in S1 File). Similar to the lgDel analyses, top-ranked clusters included terms related to glycoproteins, extracellular proteins, transmembrane proteins, homeobox proteins, and transcription. We also observed strong enrichment of a cluster of terms related to cell cycle, centromeres, and kinetochores, which we did not see in the lgDel results. Additionally, we analyzed the H9-smDel dataset and found similar candidate genes and many similar enrichment clusters (e.g., extracellular proteins, glycoproteins, homeobox proteins, transcription). Unlike the CT2-smDel analysis, terms related to cell cycle were not significantly enriched and instead, terms related to ribosome biogenesis and translation were strongly enriched, suggesting that these processes are not consistently disrupted in cell lines with a PWS genetic defect. (For complete H9-smDel results, see Tables S1, S13, and S14 in S1 File).

Finally, we further validated our findings by repeating the method on human gene expression data from neurons derived from Dental Pulp Stem Cells (DPSCs) of children with PWS and control subjects ([14]; GEO ID: GSE178687). We identified 824 candidate genes from this dataset, of which 227 genes overlap with those identified from the CT2-lgDel dataset (Fig 4C). This overlap was significantly higher than expected by chance (Fisher’s Exact Test on overlap: Odds Ratio: 2.88, p-value < 2.2e-16), further confirming the robustness and reliability of our approach (Table S11 in S1 File). In addition to identifying overlapping genes, we also compared the enriched functional clusters between the two datasets. The secondary dataset revealed 22 clusters with enrichment scores > 2 (Table S12 in S1 File). The top clusters were again similar to those in our other analyses and included glycoproteins, secreted proteins, and homeobox proteins. The significant overlap suggests that these clusters are common across PWS datasets and may provide key insight into previously unexplored disease-relevant networks.

## Discussion

### A network embedding approach to discover genes/proteins dysregulated in PWS

In this study, we overlaid PWS and healthy control RNAseq data as edge weights on two distinct human PPI networks, derived vector representations (deterministic embeddings) of protein nodes in each network, and calculated the distance between the embeddings of each node in the PWS and control networks. We interpreted that proteins with large distance values are dysregulated in PWS. Our approach is an adaptation of GeneEMBED [21]. The major difference between GeneEmbed and our work, other than the disease of interest, is that GeneEmbed based network edge weights on the predicted functional consequences of protein variants, while we based our edge weights on gene expression levels. This work underscores the utility of GeneEmbed, showing that the method can be extended to different data types and different diseases. Moreover, while there have been multiple studies on differential gene expression resulting from PWS and PWS-like genetic aberrations [11,23,28], our study is the first, to our knowledge, to use a network-based approach to analyze how those transcriptional changes are predicted to propagate through PPIs and indirectly affect other proteins.

We identified proteins dysregulated in PWS (candidate proteins) in two distinct cell lines engineered to carry a deletion within the PWS region on chromosome 15 (CT2-lgDel and H9-lgDel, Tables S2 and S5 in S1 File), two cell lines with a smaller deletion targeting the SNORD116 cluster (CT2-smDel and H9-smDel, Tables S9 and S13 in S1 File), and in neurons derived from DPSCs from individuals with PWS (Table S11 in S1 File). There was a highly significant overlap in the candidate proteins from all analyses (Fig 4D). Candidate lists included proteins encoded in the PWS deletion region as well as proteins that were consistently transcriptionally dysregulated in PWS [23]. Importantly, the top enriched functional terms obtained from DAVID analysis were shared across all candidate lists. These included terms related to glycosylation, classes of proteins that are typically glycosylated (extracellular proteins, transmembrane proteins, ion channels), and homeobox domain-containing transcription factors (Tables 1 and 2, Tables S6, S7, S10, and S12 in S1 File). The consistency of our results demonstrates the robustness of this network-based approach and suggests that our findings are not dataset-specific but have broader applicability to PWS.

Gilmore et al. [23] emphasized the importance of using isogenic controls to minimize noise in RNAseq differential gene expression analysis of PWS cells. We observed that our network analysis was also sensitive to noise in RNAseq data and that optimal results were obtained with isogenic datasets. In our analyses of cells with a large deletion in the PWS and isogenic controls (CT2-lgDel and H9-lgDel), the number of proteins exceeding the outlier threshold distance in the Control/Control comparison was relatively low (Table S1 in S1 File), and there were few proteins in common in the lgDel/Control and Control/Control outlier lists (false positives). In contrast, in our analysis of DPSCs from unrelated donors, the numbers of Control/Control outliers and false positives were much higher. Despite the noise in the dental pulp data, we observed overlap with the isogenic analyses in candidate genes and enriched functional terms, indicating that the PWS-specific signal was still detectable in the data. We conclude that genetically controlled gene expression data is essential for successful network embedding analysis, although data from genetically unrelated individuals can be useful for corroboration of results.

### Glycosylation defects may contribute to the pleiotropic phenotype of PWS

Functional enrichment analysis revealed an overabundance of glycosylated proteins amongst the candidate proteins. Abnormal glycosylation activity has been proposed as a potential contributor to the biological phenotype of PWS [29]. While no abnormalities have been detected in the glycosylation of transferrin in individuals with PWS, suggesting that N-glycosylation pathways are largely intact [29], two studies noted hyposialylation of serum apolipoprotein C‐III (apoC‐III), which is frequently observed in congenital disorders of mucin-type O-glycosylation, in a subset of individuals with PWS [29,30]. In keeping with this finding, 12 of the 39 glycosylation enzymes that are candidate proteins in CT2-lgDel and/or H9-lgDel play a role in O-glycosylation (Fig 5A). The candidate proteins also include several mucins that are tightly linked to these glycosylation enzymes in the PPI network (Fig 6A). Recently, it was reported that deletion of CHST4, one of the candidate glycosylation enzymes in both CT2-lgDel and H9-lgDel, led to obesity and intestinal inflammation in mice [31]. In this mouse model, loss of CHST4 prevented sulfation of mucins in the intestinal lining and had adverse effects on the gut microbiome. In addition, excess mucins have been detected in the saliva of individuals with PWS, which may contribute to altered saliva viscosity and dental problems [32].

Several candidate glycosylation enzymes are fucosyltransferases (Fig 5B). Individuals with congenital disorders of fucosylation have some phenotypes that overlap with PWS, including intellectual disability, hypotonia, feeding difficulties, hip dysplasia, strabismus, nystagmus, and short stature [33,34].

A third category of glycosylation enzymes represented in the candidate proteins are enzymes involved in the synthesis of glycosaminoglycans (GAGs), specifically heparan sulfate and hyaluronan (Fig 5C-D, 6C). Heparan sulfate, a sulfated GAG found on the cell surface and in the extracellular matrix, has been implicated in axon guidance during nervous system development [35]. Mutations in the heparan sulfate 6-O-sulfotransferase 1 (HS6ST1) gene were found in families with idiopathic hypogonadotropic hypogonadism (IHH), a disorder of secondary sexual development that is caused by defects in gonadotropin-releasing hormone neurons in the hypothalamus [36]. This phenotype is reminiscent of the hypogonadism and delayed or absent puberty in people with PWS [37]. Hyaluronan, a non-sulfated GAG, is a major component of the extracellular matrix in connective tissue [38] and the synovial fluid in joints [39]. Two hyaluronan synthases, HAS1 and HAS2, are candidate proteins and have close relationships with collagens and signaling molecules (e.g., MAPK3, TGFB1) in the PPI network (Fig 6C). There is currently no known link between hyaluronan and PWS, although individuals with PWS do have bone and joint abnormalities such as osteoporosis, scoliosis, and joint laxity [40].

Finally, we identified two enzymes involved in glucuronidation of steroid hormones, including sex hormones [41,42] as candidate proteins (UGT2B7 and UGT2B15; Fig 6B). Polymorphisms in the UGT2B7 gene are associated with variations in testosterone levels in men [43].

Taken together, our results suggest that PWS may be associated with abnormalities in multiple glycosylation pathways (mucin-type O-glycosylation, GAG biosynthesis, and glucuronidation) that are in turn, associated with a variety of PWS phenotypes. Therefore, it might be worthwhile to conduct molecular studies to confirm these predicted changes in glycosylation in models of PWS.

### Potential dysregulated activity of master transcription factors that drive neuronal development in PWS

Candidate lists from all of our analyses were also highly enriched for proteins with homeobox domains (Tables 1 and 2, Tables S6, S7, S10, S12, S14 in S1 File). Almost none of the proteins in the homeobox cluster were glycosylated, indicating that this cluster was distinct from the glycosylation-related clusters discussed above. It is estimated that there are about 235 homeobox domain proteins in humans, many of which are transcription factors that play a role in embryonic development [44]. Multiple subfamilies of homeobox proteins (Otx, Emx, Dmbx, Gbx, En and Hox) are implicated in the development of the brain and nervous system [45]. We found 95 homeobox proteins (~40% of total homeobox proteins) were candidates in the CT2-lgDel and/or H9-lgDel analysis; of these 61 were annotated with a role in nervous system development. Members of all of the neuronal homeobox subfamilies were represented among the candidate proteins. Given the wide-ranging neurodevelopmental phenotypes in PWS, it is plausible that abnormalities in fundamental developmental regulators like homeobox proteins play a role.

Moreover, the homebox candidates were enriched for proteins involved in the development of two neuron types–GABAnergic neurons and dopamineric neurons–that have been specifically linked to PWS. GABAnergic neurons release the neurotransmitter gamma-aminobutyric acid (GABA), the predominant inhibitory neurotransmitter in the brain. GABA signaling plays a crucial role in regulating neuronal excitability and maintaining the balance between excitation and inhibition in the nervous system. Reduced GABA levels have been observed in individuals with PWS who have more severe behavioral symptoms, including temper outbursts, skin picking, and depression [46]. Further implicating GABA signaling in PWS, a study showed significant reductions of GABA receptors bearing benzodiazepine binding sites in limbic and frontal cortical regions of individuals with PWS [47]. Three GABA receptor subunits–GABRA5 (Dental Pulp candidate protein), GABRB3, GABRG3–are encoded within the PWS locus [24], and multiple GABA receptor subunits were candidate proteins in all of our analyses. Altered GABA receptor composition may account for neurobehavioral abnormalities in PWS, including poor impulse control and impaired responses to somatic pain. Dopamine, produced by dopaminergic neurons, is a neurotransmitter involved in reward and motivation. Several studies have suggested that abnormalities in the dopamine reward system drive the insatiable appetite that is characteristic of PWS [48–50].

Finally, necdin, a protein encoded in the PWS region on chromosome 15, has been shown to activate transcription of the gonadotropin-releasing hormone (GnRH) gene in hypothalamic neurons by antagonizing MSX, a homeobox transcriptional repressor that was a candidate protein in CT2-lgDel [51]. Loss of necdin due to deletion or suppression of the PWS locus, leading to persistent repression of the GnRH gene by MSX, could contribute to hypogonadism in individuals with PWS.

### Conclusions and future work

Prior studies on PWS largely analyze genes in isolation without the broader context of protein interactions. By focusing on how expression changes in the PWS dataset reverberate through the PPI network, we identify key proteins that are functionally affected in PWS and may play a bigger role in the condition than originally thought. Given that our findings implicate both proteins associated with PWS and proteins not previously linked to PWS, this suggests our approach uncovered functionally relevant genes that traditional methods have overlooked.

Protein glycosylation and transcriptional dysregulation by homeobox domain proteins both emerged through the network analysis as novel pathways not previously linked to PWS, yet strongly aligned with known disease features. Glycosylation abnormalities could contribute to PWS-related phenotypes such as altered saliva composition, gut inflammation, and hormone imbalances, while dysregulation of homeobox transcription factors could contribute to PWS-related neurodevelopmental defects such as impaired GABAergic and dopaminergic signaling, behavioral issues, hyperphagia, and hypogonadism. Although experimental validation is still needed, our findings provide strong candidates for further investigation and open new avenues for understanding PWS. The consistency of these findings across diverse neuronal models highlights the robustness of the approach and its potential to reveal disease-relevant biology across datasets.

Finally, we have demonstrated that our adaptation of the GeneEmbed method for network embedding-based disease discovery is effective at uncovering proteins important in PWS. Our work can serve as a framework for identifying key proteins and pathways that are hidden from traditional omics analysis methods and can be adapted to different diseases and omics data types. This is critically important in rare diseases like PWS where experimental studies on molecular mechanisms may be scarce.

## Materials and methods

### Overview

Fig 1 illustrates an overview of the workflow and analysis pipeline, which was adapted from GeneEMBED [21]. The goal was to identify proteins that might play an important role in PWS due to indirect effects of the propagation of gene expression changes through a PPI network. For each analysis, we constructed two weighted human protein-protein interaction networks, one with edge weights calculated using gene expression data from PWS cells and the other with edge weights calculated using gene expression data from control cells. Aside from the edge weights, the two networks had identical structures. Next, we used the GraphWave algorithm that uses network connectivity and edge weights to calculate embeddings for the nodes (proteins) in each network. We performed dimension reduction on the embeddings using Principal Component Analysis (PCA) and then calculated the distance between the PWS and control embeddings for each protein. Proteins with large distances were assumed to be strongly affected in PWS and were selected as candidate genes. Finally, we performed functional enrichment analysis on the candidate proteins to discover biological functions and processes that may be disrupted in PWS. Details of the method are provided in the following sections.

### Datasets

We analyzed two publicly available PWS RNAseq gene expression datasets. The first was generated using isogenic human embryonic stem cells that were differentiated into neurons [23]. Two cell lines, H9 and CT2, were engineered with CRISPR to create specific deletions on the paternal chromosome 15q allele (see Gilmore et al. [23] for more details). The large deletion (PWS-lgDel) model removed the region from the alternative promoters of the SNRPN transcript upstream of the PWS-IC to the distal end of the SNORD116 snoRNA cluster. The small deletion (PWS-smDel) model targeted only the *SNORD116* cluster. For each cell line, six replicates were available for each group (lgDel, smDel, and Control (no deletion)). We performed four comparisons using these datasets: i) CT2-lgDel vs. CT2-Control; ii) H9-lgDel vs. H9-Control; iii) CT2-smDel vs. CT2-Control; iv) H9-smDel vs. H9-Control. The second dataset was generated from dental pulp stem cells (DPSC) that were differentiated into neurons [14] (GEO ID: GSE178687). Data from twelve subjects with PWS were available: four with chromosome 15 deletions, four with chromosome 15 maternal uniparental disomy (UPD) and autism spectrum disorder (ASD), and four with UPD without ASD. Each PWS subgroup was compared to four matched control subjects and the results were averaged.

### Gene expression data processing

For each sample group, gene expression values were aggregated by taking the average read count value of the replicates. The aggregated expression values were transformed using a base-2 log scale to minimize the magnitude of differences between genes with low and high expression levels. This transformation helps prevent highly expressed genes from disproportionately dominating the analysis. For genes with zero counts in all replicates, which cannot be directly log-transformed, we assigned a floor value of 0.125 before applying the base-2 log transformation.

### Human protein-protein interaction network

The PPI networks were created by querying the STRING database for genes with a confidence score of 0.4, a medium confidence score, to avoid false positive interaction while not being too stringent [22]. The STRING confidence score estimates the probability that a predicted protein–protein association is biologically meaningful, based on combined evidence from multiple independent sources, capturing both physical and indirect functional associations. Interactions are determined from five evidence channels: Genomic Context Predictions (including gene neighborhood, gene fusion, and phylogenetic co-occurrence), High-throughput Lab Experiments, Co-Expression, Automated Text Mining, and Previous Knowledge in Databases. The base PPI network has 16,826 nodes and 770,500 edges. The confidence score of 0.4 was used for selecting protein interactions to include in the base networks and did not contribute to the calculations of edge weight.

### Edge weight calculation

After processing the expression data and establishing the base PPI network, we integrated gene expression values to create sample group-specific weighted networks (e.g., one network for CT2-lgDel and one network for CT2-Control). Gene expression values were mapped to their corresponding protein nodes using gene identifiers, and used exclusively to compute edge weights. The STRING network topology was kept fixed across conditions with expression data used only to define condition-specific edge weights. For each interaction edge in the network, we calculated weights using the sum of the mean and minimum expression values of the two interacting proteins. This approach ensures that the edge weight reflects both the overall expression level (mean) and the potential limiting factor (minimum) in the protein interaction. The resulting edge weights were then normalized to a scale of 0–2.

### Node embedding

We used the GraphWave algorithm to generate embeddings for each node in the PPI network [20]. GraphWave captures the topological structure of a network by leveraging spectral graph wavelet analysis. It models how diffusion processes propagate from each node across the network and represents the resulting pattern as a vector to capture both local and global structural properties. GraphWave was chosen over other embedding algorithms due to being deterministic, i.e., given the same network, the algorithm will generate the same embeddings every time it is run. This choice was motivated by our desire to compare embeddings of the same node (protein) across networks that had the same structure but differing edge weights. This enables direct comparison of node embeddings across conditions, such that differences reflect changes in edge-weight–induced network context rather than random variation. To validate the algorithm’s determinism, we ran it multiple times on the same network, consistently producing identical gene embeddings. The algorithm was set to generate 100-dimensional embeddings for each node (protein).

### Dimension reduction and node distance calculation

The 100-dimensional embeddings generated by GraphWave capture a wide range of information, from local neighborhood relationships to global structural properties of the graph. This extensive scope means that the embeddings can be sensitive to changes in edge weights across the entire graph. Therefore, we employed Principal Component Analysis (PCA) to reduce the noise in the high-dimensional embeddings. We focused on the first and second principal components as they capture the most significant variations, accounting for 90–95% of the overall variance. Then, for each protein, we calculated the Euclidean distance between its dimension-reduced embeddings in the PWS network and the corresponding control network.

### Outlier detection

We identified outlier genes using the Interquartile Range (IQR) outlier method: genes with a distance greater than 1.5 x IQR were flagged as outliers. The outlier genes represent proteins whose embeddings exhibit the greatest sensitivity to differences in gene expression between the PWS group and control group. The outlier genes were subjected to false positive testing using control-control analysis (details are discussed below) to filter disparities that are not PWS-specific, ensuring the remaining outliers represent PWS-specific network changes.

### False positive analysis

To filter out false positive proteins (i.e., proteins with high Euclidean distances that are not specific to the disease state), we implemented a Control-Control comparison using the same network analysis pipeline. We split the control replicates into two groups (Control-A and Control-B), averaged the expression values in each group, and applied these as edge weights to the base PPI network used in PWS-Control analysis. Then we performed the same embedding and distance calculations as in the PWS-Control analysis. Control-Control outlier genes were identified using the 1.5 x IQR threshold established from the PWS-Control analysis. Genes classified as outliers in both analyses were considered false positives. The PWS-Control outlier list was then filtered to remove any false positives, and the remaining proteins were considered the candidate genes.

### Enrichment analysis

We analyzed the candidate genes with the enrichment analysis tool Database for Annotation, Visualization, and Integrated Discovery (DAVID) to identify enriched annotation terms [52]. Using DAVID’s default settings, terms with Benjamini-Hochberg corrected p-values < 0.05 were considered significant. Enriched terms were grouped into clusters using the Functional Clustering Analysis tool in DAVID.

### Network visualization

Networks were visualized using Cytoscape (v3.10.3) [53]. PPIs among an input list of proteins were retrieved from STRING using the Cytoscape stringApp (v2.2.0) [54] in “protein query” mode with a confidence threshold of >0.4. Enriched functional terms in the networks were identified using the STRING Enrichment tool that is built-in to stringApp. Terms were considered to be enriched if they had an FDR-corrected p-value < 0.05.

### Gene expression heatmaps

Heatmaps of gene expression levels were generated using the package pheatmap (v1.0.12; https://github.com/raivokolde/pheatmap) in R (v4.4.1). Expression levels were taken from Gilmore et al. [23] and were z-scored by row.

## Supporting information

S1 Fig Heatmaps showing gene expression levels of glycosylation enzymes in (A) CT2-lgDel and (B) H9-lgDel. Gene expression data is taken from PMID: 39575480. Expression levels were z-scored by row.
(PDF)

S2 Fig Network showing protein-protein interactions among CT2-lgDel candidate glycosylation enzymes and their CT2-lgDel candidate first neighbors. Nodes with black borders are glycosylation enzymes that are CT2-lgDel candidates; nodes with red borders are glycosylation enzymes that are both CT2-lgDel and H9-lgDel candidates. Node fill color indicates Euclidean distance between lgDel and control networks.
(PDF)

S1 File Supplementary Tables S1-S14. (XLSX)
