# Supplementary figures and images for: A network-centric approach reveals novel pathways impacted by Prader-Willi Syndrome
Source: PLoS One. 2026 Apr 28;21(4):e0347773. doi: 10.1371/journal.pone.0347773 (PMC13123929; doi:10.1371/journal.pone.0347773)

A

CT2

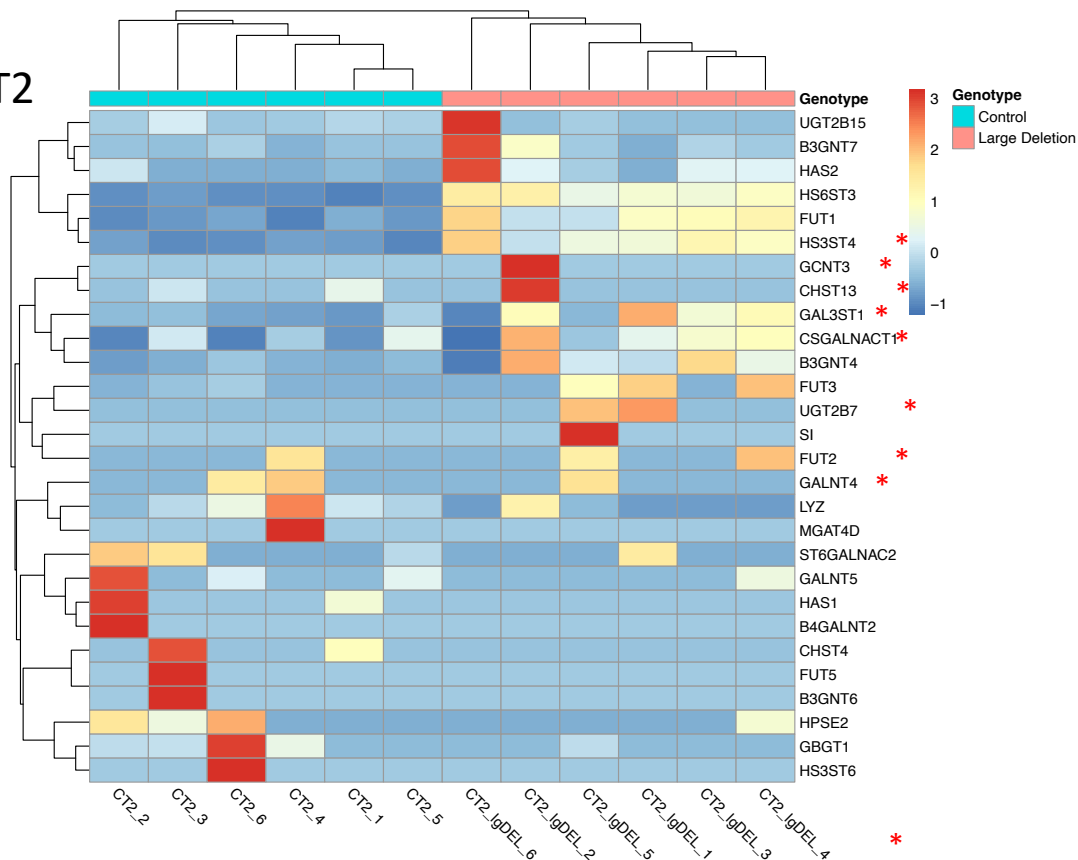

B

H9

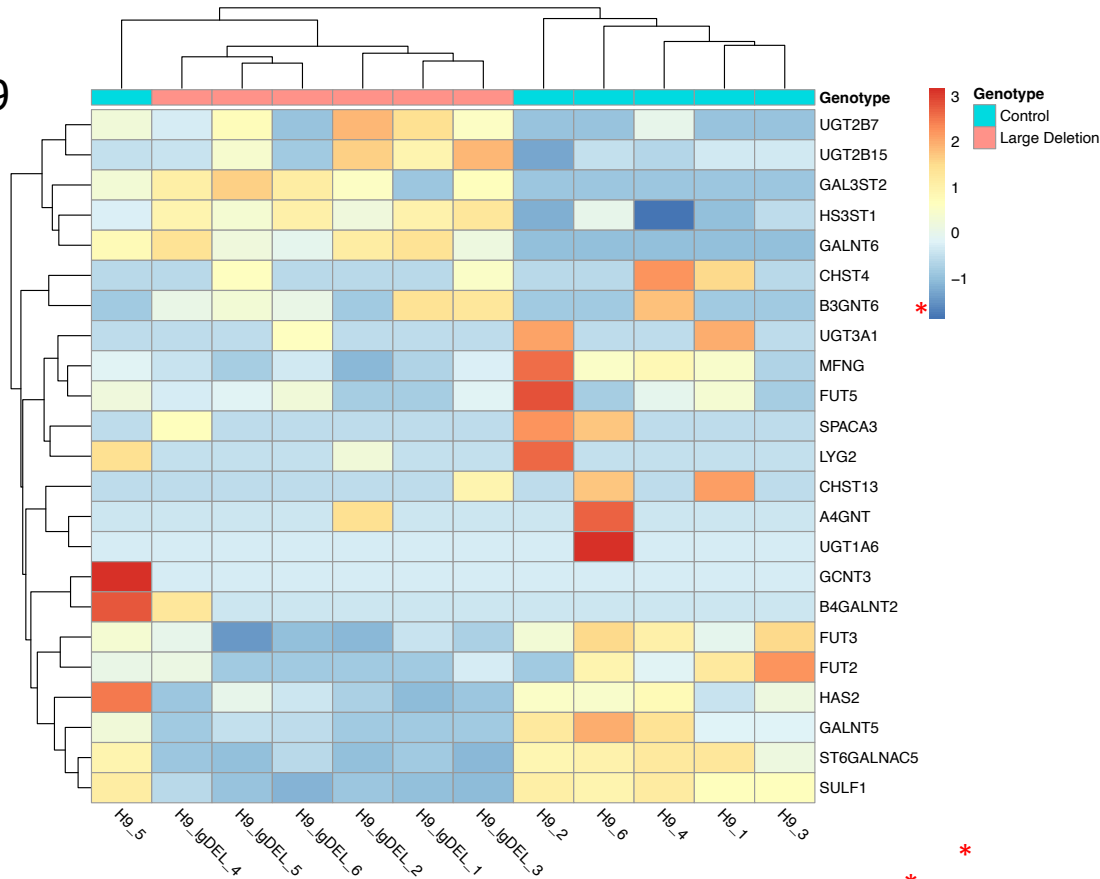

Supplement: S1 Fig — Gene expression data is taken from PMID: 39575480. Expression levels were z-scored by row. (PDF) [file pone.0347773.s001.pdf]
