# Supplementary material for: A network-centric approach reveals novel pathways impacted by Prader-Willi Syndrome
Source: PLoS One. 2026 Apr 28;21(4):e0347773. doi: 10.1371/journal.pone.0347773 (PMC13123929; doi:10.1371/journal.pone.0347773)
Supplement: S2 Fig — Nodes with black borders are glycosylation enzymes that are CT2-lgDel candidates; nodes with red borders are glycosylation enzymes that are both CT2-lgDel and H9-lgDel candidates. Node fill color indicates Euclidean distance between lgDel and control networks. (PDF) [file pone.0347773.s002.pdf]

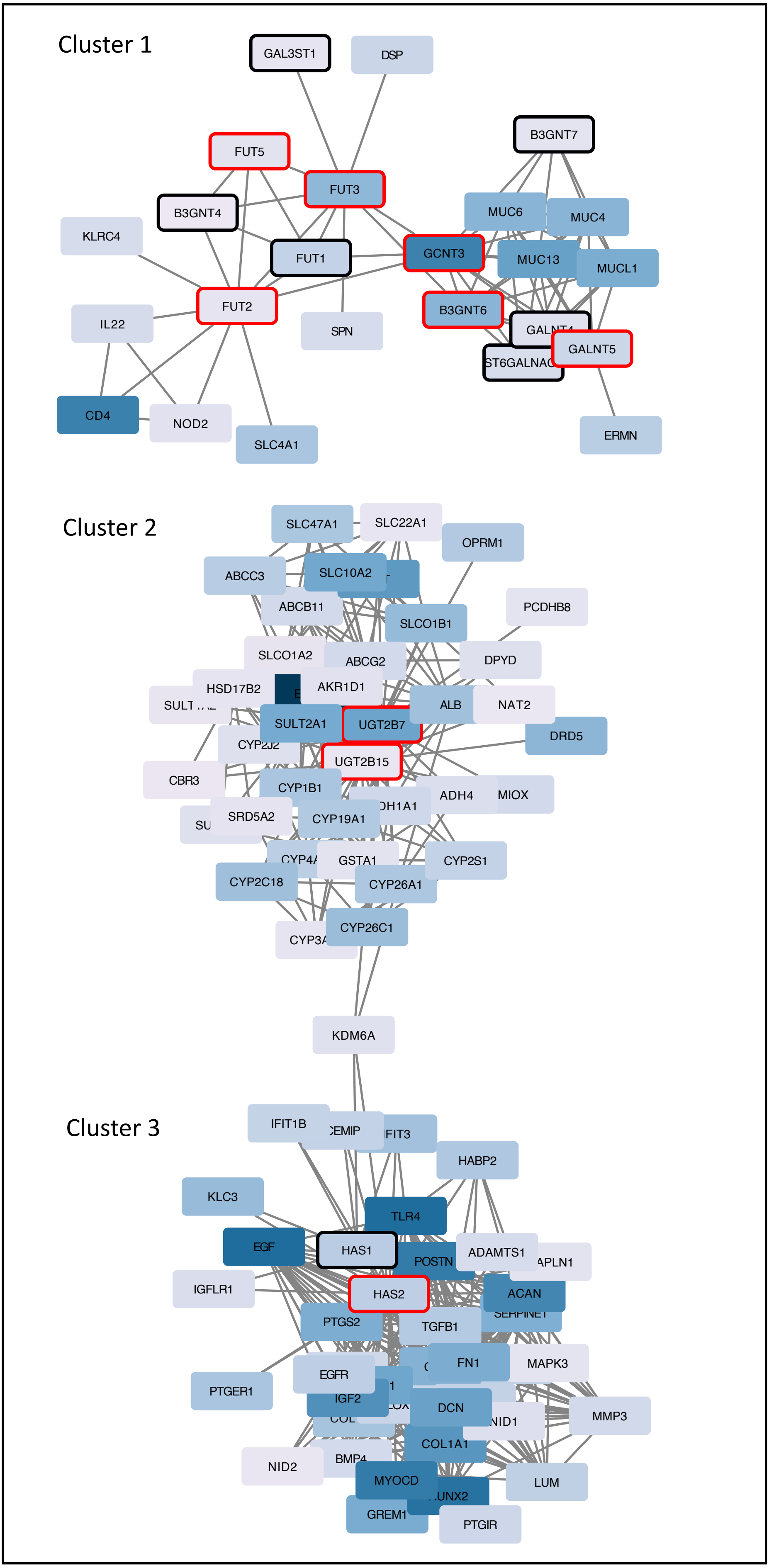
Cluster 1
GAL3ST1
DSP
FUT5
FUT3
B3GNT7
B3GNT4
MUC6
MUC4
KLRC4
FUT1
GCNT3
MUC13
MUCL1
FUT2
B3GNT6
IL22
SPN
ST6GALNAC
GALNT5
CD4
NOD2
SLC4A1
ERMN
Cluster 2
SLC47A1
SLC22A1
OPRM1
ABCC3
SLC10A2
ABCB11
PCDHB8
SLCO1B1
SLCO1A2
ABCG2
DPYD
HSD17B2
AKR1D1
ALB
NAT2
SULT2A1
UGT2B7
DRD5
UGT2B15
CBR3
CYP1B1
ADH4
MIOX
SRD5A2
CYP19A1
GSTA1
CYP2S1
CYP2C18
CYP26A1
CYP26C1
KDM6A
Cluster 3
IFIT1B
HABP2
KLC3
TLR4
EGF
HAS1
POSTN
ADAMTS1
IGFLR1
HAS2
ACAN
PTGS2
TGFB1
FN1
MAPK3
EGFR
PTGER1
IGF2
DCN
MMP3
COL1A1
NID2
BMP4
MYOCD
LUM
GREM1
PTGIR
